# Medical error reporting software program development and its impact on pediatric units’ reporting medical errors

**DOI:** 10.12669/pjms.36.2.732

**Published:** 2020

**Authors:** Aysun Unal, Seyda Seren Intepeler

**Affiliations:** 1 Dr. Aysun Unal, PhD, RN. Assistant Professor, Nursing Management Department, Akdeniz University Kumluca, Faculty of Health Sciences, Antalya, Turkey; 2 Prof. Dr. Seyda Seren Intepeler, BSN, PhD. Nursing Management Department, Dokuz Eylul University, Nursing Faculty, Izmir, Turkey

**Keywords:** Culture of safety, Medical errors, Patient safety, Quality improvement, Reporting system

## Abstract

**Objective:**

The purpose of this quasi-experimental study was to developing web-based, anonymous reporting system to increase reporting of medication errors, blood transfusion errors and patient falls in pediatric units and to compare the computerized system with the written system already in use at the institution.

**Methods:**

This study was conducted in all pediatric units of a research hospital. All physicians and nurses working in these units agreed to participate in the study. All units were visited to introduce the new reporting system. The number and quality of the reports sent on the new system in years 2014 and 2015 were compared to the reports sent the previous year using the written system.

**Results:**

There was considerable increase in rates of reporting: 234% increase in medication error reporting rate, and 100% increase in the reports of blood transfusion errors. One of the most important results of this study that near-miss errors were not reported at all while the written system of the study institution was being used, whereas it was the most commonly reported type of errors in the electronic error reporting system.

**Conclusion:**

The web-based reporting system, which makes reporting easy, promoted the development of safety culture among doctors and nurses in common language.

## INTRODUCTION

Medical error is defined as “failure of a planned action to be completed as intended or use of a wrong plan to achieve an aim”.^1^ A medical error is a threat to patient safety and has a negative effect on health as well.^2^ On a global scale, it is estimated that 94,000 people died in the year 1990 as a result of medical errors; this increased to 142,000 people in 2013.^3^ A literature review evaluating the numbers of lethal and serious medical errors found that annually 210,000 deaths at hospitals are related to preventable harm.^4^ Medical errors related to medication administration and blood transfusion, as well as falls—which are included as medical errors—are in the forefront in pediatrics due to the unique characteristic of children. Errors related to medication administration are the most common type of medical error in pediatric units. Medication errors, a worldwide problem for hospitals, involves all negative events that occur during the supply of medications, their administration to the patients and monitoring patients.^5^ Miscalculating the dosage of medication is a serious security issue that threatens children under treatment because it is complicated: tiny dosage errors may cause serious damage harm.^6^

Health professionals working in pediatric units should expand efforts to eliminate these preventable these medical errors, and should adopt a safety culture that supports that effort.^7^ One of the strategies in safety culture is establishing and maintaining proper reporting systems. Reporting systems are beneficiary in analyzing the processes for both factors causing errors and precautions should be taken to prevent them.^8^ Considering that there is a significant lack of reporting medical errors, the cost regarding human life and financial resources can be quite high.^9^

Despite the importance of error reporting, there are many barriers that prevent the health professionals from reporting errors.^10^ The relevant literature focuses on the importance of anonymous reporting systems that are addressed to increasing the number of reports by eliminating barriers that interfere with reporting errors by health professionals.^11^

Examination of various web-based reporting systems developed around the world shows that there are specific reporting systems such as anonymous, web-based standardized monitoring techniques^12^ and medication error reporting systems^13^, Electronic Clinic Safety Reporting System, which are developed for hospitals.^14^ Users enter texts about what happens during the reporting, which are not configured, as well as the classified and coded structures of data. These texts have the extensive information related to the error, but it is not easy to make inquiries into uncoded data. Therefore, the manual interpretation of data can produce variable results depending on the individual.^15^

The comparison of these systems shows that they have differences in terms of scope and classification, and they should be used in every care environment. Different software needs to be developed with common terminology for the interpretation of data in the systems. In this respect, it is clear that different designs and software should be compared to develop evidence-based web-based reporting systems and a common software language that can be used in all care environments.

The present study focused on developing a web-based, anonymous reporting system to increase reporting of medication errors, blood transfusion errors and patient falls in pediatric units. Unlike other systems, the software of the system was written based on a common terminology including the classifications, severity and outcomes of errors by encoding each error type within itself to minimize the interpretational differences in manual entries under the guidance of new and updated suggestions in the literature.

## METHODS

### Study Design

This quasi-experimental study that was conducted in all pediatric units of a research hospital. This clinic is pediatric emergency, pediatric intensive care, newborn intensive care, pediatric psychiatry, pediatric surgery, pediatric hemato-oncology, general pediatrics. In all, 72 physicians and 77 nurses worked in these clinics during the study; all of them agreed to participate in the study.

All units were visited to introduce the new reporting system. The researcher provided practical training in these clinics on the reporting of events. The number of the reports sent on the new system in years 2014 and 2015 were monitored and compared to the reports sent the previous year using the written system.

### Strategies Used in The Development Process, and the System Software

Error reporting barriers restrict a close monitoring of events and making improvement in patient safety.^16^ Therefore, the present researcher created strategies in the first stage of system design addressed to the reporting barriers stated in previous literature.^17^,^18^ Considering this barriers, the researcher decided to make the system anonymous and voluntary, time saving and providing quick reporting and to provide open access on a general web server. However, the researcher decided to code all error-related desired data to the system to include the information that would enable root-cause analysis, patient outcomes and patient categorizations. The Patient and Employee Safety Regulations prepared by Turkish Ministry of Health require that events that must be reported at a minimum level include adverse medication, blood transfusion errors and falls events.^19^ Thus, this study determined that these three categories should be examined. Since the types, causes and outcomes of these categories were different from each other, a comprehensive database was developed so that it would be informative for users. The researcher used standardized opening buttons for each error type, and used standard boxes again after each box is opened for the error date, shift, error description and reasons for the error. Selection lists included the sub-reasons for the relevant error; these lists appeared when the users clicked on any event reason. The user could then see the sub-reasons of the event and select from them. For medication and blood-transfusion related errors were standardized by reference to the literature.^20^,^21^ As in the literature, the medication and transfusion errors within the scope of medication and blood transfusion safety involve events that occur in all processes from the production of the medications or blood products to the monitoring interval after their administration in Turkey. In reports related to falls, the researcher provided standardization by combining all pediatric fall assessments and risk scales in the literature.^22^ Applying all these considerations, the researcher created an integrated system structure that combined multiple areas, helped viewing the data accurately, enabled users to make notifications by clicking rather than typing and allowed system access on smartphones and tablets as well.

## RESULTS

### The Numbers and Types of the Reported Errors

**Fig.1:**
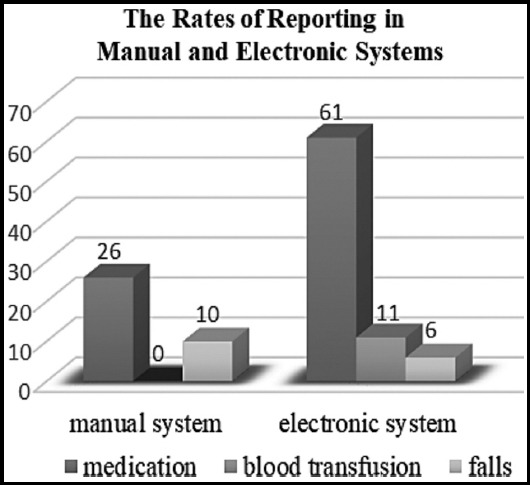
The distribution of the reports on the old and new systems.

A comparison of the rates of reporting on written and electronic-based error reporting systems in pediatric units revealed that the reporting rates this year increased by 216% compared with the number of reports sent on the written system the previous year (Fig.1).

An analysis of the medication errors on the web-based system by their types showed that the highest number of errors reported were related to incorrect calculation and administration of dosages during the stage of initial prescription by the physician. The least number of errors were reported related to the administration of the medication to the wrong patient (Table-I).

**Table-I:** The distribution of medication errors by their types.

Errors	n	%
Incorrect dosage	44	70.4
Wrong medication	6	9.8
Incorrect time	4	6.5
Skipped doses	3	4.9
Monitoring error	3	4.9
Wrong patient recipient	2	3.2

Total	62	100

Among the blood and blood products transfusion errors, 45.4% included an incorrect calculation of the transfusion amount when the physician ordered transfusion and 18.5% included allergic reactions and similar events (Table-II).

**Table-II:** The distribution of blood and blood products transfusion cases by case type, product qualities, and age (n=11).

Case Type	Blood Product	Age	%
Excessive transfusion	Human albumin	1	9
Higher amount of transfusion	Erythrocyte suspension	1	
Higher amount of transfusion	Erythrocyte suspension	5	
Lower amount of transfusion	Erythrocyte suspension	2	45.4
Higher amount of transfusion	Erythrocyte suspension	2	
Higher amount of transfusion	Erythrocyte suspension	1	
Immunological hemolysis due to alloantibody	Erythrocyte suspension	2	18.5
Immunological hemolysis due to ABO incompatibility	Erythrocyte suspension	1	
Anaphylaxis /excessive sensitivity red cells Anaphylaxis	Fresh frozen plasma	8	18.5
	Fresh frozen plasma	3	
Blood product being prepared with missing ingredient (without apheresis)	Thrombocyte suspension	14	9

Total		11	100

Of the six fall errors that were reported, two were near-miss events. Falls were mostly caused by environmental factors (66.6%). The evaluation of fall errors indicated that one error caused medium-level damage to the patient (Table-III).

**Table-III:** The distribution of falling reports by type, causal factors, patient outcomes, and age (n=6).

Type of Case	Factor	Patient Outcomes	Age	n	%
Near-miss of Fall	Parent Participation	No harm	7	2	33.3
	Age	No harm	3		
Fall	Environmental Factors	Medium level Damage	4	4	66.6
	Environmental Factors	No harm	1		
	Environmental Factors	No harm	2		
	Environmental Factors	No harm	1		

### The Reasons of the Reported Errors and Patient Outcomes

The study evaluated the reasons for the medication and blood transfusion errors reported on the electronic reporting system and found that the highest number of reports involved incorrect calculation of dosages or infusion rates (44.4%). The lowest number of reports involved on the system were incorrect labeling and packaging (4%) (Table-IV).

**Table-IV:** The Distribution of the Factors That Caused the Reported Errors (n=72).

Reason	n	%
Incorrect calculation of dosage or amount of infusion	32	44.4
Lack of knowledge	12	16.6
Communication	8	11
Stress and heavy workload	7	9.7
Confusion of names	5	6.9
Problems related to the storage and delivery of blood	3	4
Incorrect labeling and packaging	3	4
Incorrect computer entries	2	2.7

In the patient outcomes categories of errors, It was determined that the near-miss events had the highest rate (56.9%) among the reported errors. Analysis of other patient outcomes showed that the rate of real events that affected the patients was 43%.

## DISCUSSION

The most important result obtained by this study was that a web-based system that is voluntary and facilitates reporting was used by the doctors and nurses effectively at the same level and that a common terminology was created between team members during the process of reporting. Since the most important indicator of safety culture is that all of the team members use a common terminology on a common platform to prevent errors, the web-based reporting systems positively affected the development of safety culture. A significant increase in reporting rates as a result of the web-based reporting system.. Consistently, previous studies managed to increase reporting rates when the reporting was anonymous and performed on web-bases systems.^11^,^12^,^15^

An evaluation of the rates of the reported errors showed that medication errors were the most frequently reported events. The types and frequencies of medication errors indicated that dosage calculation errors were common The complicated calculations of small dosages for children increase the occurrence rates of these errors.^6^ The researcher believes that these errors are reported more frequently because medication errors are more common in pediatric units.

The blood transfusion errors that were reported on the web-based system had not been reported on the previous written system. This is probably due to the lack of knowledge and awareness among staff about the blood transfusion-related errors that should be reported. The study evaluated blood transfusion errors and found that the reported errors most frequently were unnecessary transfusion, calculation of the transfusion rate higher than necessary, and allergic reactions. The relevant literature also indicates that the most common errors in pediatric units were excessive transfusion and lack of knowledge about special needs of newborns and reactions to transfusion.^23^ There were no direct study results related to the reporting of blood transfusion errors.

The present study also analyzed the fall events reported and found that there was a decrease in the reporting of these events compared with the written system. At the beginning of the year when the study was conducted, the risk of falling began to be identified for each patient, and the necessary precautions were taken in the institution. It is also thought that the training concerning falling risk analysis provided in the units where the study was conducted had effects on taking necessary precautions through paying attention to the risk factors in practice. In the written system, the reports were sent on the occurrence of the falling events, whereas the computerized system involves reporting the near-miss falling events. This study pointed out the importance of reporting near-fall events, and such events began to be reported. Information obtained from reports is expected to guide institutions in the development of prevention strategies, and to contribute to the literature. This implies that it is possible to make a more comprehensive evaluation of the falling risks in children.

An evaluation of the reasons for medication, blood, and blood products transfusion errors showed that the most common errors was incorrect calculation of dosages and infusion rate in addition to the lack of information about drugs. Subsequently, communication problems, heavy workload and stress, confusion with the names of drugs, problems with the storage and delivery of blood. Many drug formulations are suitable for adults but not for children, which makes it more difficult to find and calculate the specific dosage for various age groups.^6^ In the relevant literature, there are many reasons suggested for the high rate of medication errors in pediatric units, including staff fatigue and lack of concentration due to heavy work load, and similar names given to different drugs.^24^ The patient outcomes of the errors reported in the computerized system showed that the most reported errors were the near-misses. Since the institution’s written system was suitable only for reporting the events that had occurred, near-miss events were not reported. On the other hand, the relevant literature indicates that the rate of reporting is higher when the errors result in serious harm, and there is less reporting, accordingly, when the possible results cause less or no harm to patients.^18^ The computerized system provided an awareness about the notification of near-miss events.

## CONCLUSION

After the web-based error reporting system began to be used in pediatric units, there was a considerable increase in the rates of reporting. However, this system needs to be improved along with similar reporting systems in current use. Considering these necessities, it is also suggested that repetitive tests should be administered in the software development stage of web-based reporting systems, and that the systems should be designed to enable a comparative analysis of the data (e.g. the types, causes of errors) using standardized terms to classify them. The newly designed systems should be suitable for integration with the automation systems used in health institutions, considering especially patient and employee characteristics. The web-based software should be capable notifications as reminders and supporting information, and show warning messages about incorrect computer order entries.
